# Annotations

**Published:** 1892-05-21

**Authors:** 


### ANNOTATIONS.

BRITAIN is ruled by a democracy; medical men, as medical men, are governed by an oligarchy.

**Curiosities of Médical Politics.**

In the great matters of high imperial policy every workman who pays his rates is entitled to a full vote: in the small matters of purely medical business the average medical man has a fifth part of a vote. The great world turns its schoolboys and students out of school and college, and freeing them from the tutelage of the schoolmaster, bids them play their part like men; and this freedom is given to human creatures at all ages between fourteen and twenty-four. The small medical world is never delivered from the authority of its schoolmasters at all. The General Medical Council is constituted as to four-fifths of its whole by nominees, the representatives of Universities, colleges, and other educational institutions, and as to one-fifth by elected representatives of the general body of the profession. Now the General Medical Council is the final Court of Appeal in matters medical, and is, in short, the permanent government of the profession. We have then this curious anomaly, that whilst the least educated part of the public govern the whole State, including the medical profession and the General Medical Council in questions imperial, the great body of doctors are not thought sufficiently capable to govern themselves in matters purely medical. This is undoubtedly injurious to the rank and file of practitioners. It is a common-place in economics that self-government develops governing capacity. One cannot help wondering that medical men permit themselves to be ruled throughout the whole of their lives by nominees of educational institutions. Frequent attempts have been made to assert the equality of the medical man with the farm labourer in this question of self-government; and Dr. Rentoul, of Liverpool, is once more urging the question upon the attention of the members of the British Medical Association. Dr. Rentoul points out that the greater proportion of the revenues of our legislative medical assembly is drawn from medical practitioners. Taxation and representation are correlatives in England, and one cannot help feeling a little humiliated that principles which were commonplaces in the time of Simon de Montfort are considered too visionary for application in the learned profession of medicine. So true it is, that too much tutelege results in perpetual childhood for the tutored.

IT is often said, "The nearer to church the further from grace," and an article in the *British Medical Journal* suggests the reflection that

**Why will Doctors not Save?**

the more familiar a man is with human mortality the less is he inclined to believe that he himself is mortal. The writer in the journal is quite pathetic in his despair of inducing men to save. He assures his readers that the amount of poverty and distress existing amongst the widows and orphans of medical men is very large. He also reminds them that the Registrar-General's returns prove that the lives of men in active practice are very short; and he asks pathetically: "Ought men to marry if they cannot make some provision for those they leave behind?" Some of the facts which he relates point to an excess of heedlessness and indifference on the part of medical men in this primarily important matter. For example, we are told that "the society for widows and orphans of medical men presided over by Sir James Paget has a large accumulated fund, but that only 300 or 400 medical men out of the enormous number of practitioners resident within the metropolitan area can be induced to join it." The Surrey Medical Benevolent Society also has a small but well-established fund. It has five free scholarships for boys at Epsom, and ten for girls elsewhere; and yet only forty-eight of the very large number of medical men practising in Surrey have joined its ranks. The subscription to each of these societies is only two guineas per annum. The poverty of aged medical men and their wives is painfully shown by the fact that a short time ago seventy poor aged applicants were competing for annuities of £10. On the other hand, the advantages of joining the London society for medical widows and orphans is still more strikingly evidenced by the statement that one member who died before he had paid the society so much as £20 secured for his widow and children payments extending over several years, which amounted altogether to a sum of no less than £2,000. If these facts, which are urged upon the members of the profession by the *British Medical Journal*, do not make men provident, it is much to be feared that they will not be made provident at all.

THERE is a prejudice against the ballet-girl. Many really kind-hearted people are ready to espouse

**English Ballet-Girls Abroad.**

the cause of governesses, shop-girls, and domestic servants, to see that these can obtain proper lodgings, are looked after when out of work, and helped to obtain new situations, but they do not interest themselves in the ballet-girl. They look upon her as a creature of folly, frivolous at best, and earning her living in a dubious way. Now, the majority of ballet-girls are like the majority of other working girls, who do the work they have been taught to do, and dance in the spirit of the ant rather than the grasshopper. In London they are generally well enough off; they live at home, they have such care as they need, and their earnings are sufficient for their wants. But if, tempted by an apparently higher salary they go to Paris, where the fresh faces and unstudied grace of English girls are much appreciated, they are placed in a different position. They are ignorant of the language, and ignorance of this kind always implies expense. They do not know where to find lodgings; they do not know where to dine cheaply and reasonably well; they do not know the shortest way to the theatre, and are fined because they arrive late. Meanwhile, the small stock of money which they brought with them dwindles and disappears before they receive their first pay at the end of a month's work. There are often anxious hearts behind the glittering dresses, and troubled eyes under the pencilled brows. What these girls need is a friend and a home. The friend they have in Mrs. Lewis, better known as Miss Leigh, who has befriended so many English and American girls. Mrs. Lewis asks for means to provide the home. The existing institution, of which she is Lady President, does not meet the case, for ballet-girls who leave the theatre near midnight must have their resting-place near at hand. The expense of travelling any great distance to and from rehearsal and performance would be so great an addition to their rent that a home, however good and cheap, that is not near the theatre is out of the question. Yet such a home, under the charge of some motherly Christian woman, would be an inestimable benefit. We have spoken only of its value in the matter of physical well-being, but it must be obvious that to a young girl in a strange land a home, an adviser, and a friend may mean the salvation of a soul. Without knowledge where and how to obtain the best value for her money, a salary that in England would mean comfort will, in Paris, barely keep her above starvation. Mrs. Lewis has written to the English public for money to help her in caring for their country-women in France. It is to be hoped that she will not appeal in vain. Contributions may be sent to Mr. F. A. Bevan, 54, Lombard Street; Miss Rhoda Mouncey, 22A, Queen's Road, Bayswater; or to Mrs. Lewis herself, at 77, Avenue Wagram, Paris.

